# Toward Enhanced Humidity Stability of Triboelectric Mechanical Sensors via Atomic Layer Deposition

**DOI:** 10.3390/nano11071795

**Published:** 2021-07-09

**Authors:** Wook Kim, Sumaira Yasmeen, Chi Thang Nguyen, Han-Bo-Ram Lee, Dukhyun Choi

**Affiliations:** 1 Department of Mechanical Engineering (Integrated Engineering Program), Kyung Hee University, Yongin 17104, Korea; choice124@khu.ac.kr; 2 Department of Materials Science and Engineering, Incheon National University, Incheon 22012, Korea; sumairayasmeen51@gmail.com (S.Y.); victornguyen@inu.ac.kr (C.T.N.)

**Keywords:** atomic layer deposition, surface control, humidity stability, triboelectric behavior

## Abstract

Humid conditions can disrupt the triboelectric signal generation and reduce the accuracy of triboelectric mechanical sensors. This study demonstrates a novel design approach using atomic layer deposition (ALD) to enhance the humidity resistance of triboelectric mechanical sensors. Titanium oxide (TiO_x_) was deposited on polytetrafluoroethylene (PTFE) film as a moisture passivation layer. To determine the effective ALD process cycle, the TiO_x_ layer was deposited with 100 to 2000 process cycles. The triboelectric behavior and surface chemical bonding states were analyzed before and after moisture exposure. The ALD-TiO_x_-deposited PTFE showed three times greater humidity stability than pristine PTFE film. Based on the characterization of TiO_x_ on PTFE film, the passivation mechanism was proposed, and it was related to the role of the oxygen-deficient sites in the TiO_x_ layer. This study could provide a novel way to design stable triboelectric mechanical sensors in highly humid environments.

## 1. Introduction

The expansion of personal and mobile electronic devices, and rapid developments in information and communication technology (ICT), are noticeably changing our modern civilization. These phenomenal developments have introduced a new age, called the fourth industrial revolution. In the fourth industrial revolution, there are several key technologies such as Internet of Things (IoTs), artificial intelligence (AI), big data analysis, three-dimensional (3D) printing, and augmented reality. In addition to the development of these key technologies, their integration has been processed toward smart platforms, such as a smart factory, smart healthcare, and smart city. To establish the smart platforms, it is important to obtain correct data from environments by using sensors. Therefore, the importance and necessity of efficient and precise sensors has increased.

To satisfy societal demands, various sensors based on the piezoresistive effect [1,2,3], capacitive sensing [4,5,6], and piezoelectric behavior [7,8,9] have been investigated. Triboelectric effect-based sensors have been investigated to develop reliable mechanical and chemical sensors. The triboelectric sensors can detect changes in pressure [10,11,12,13,14], mechanical motion [15,16,17,18,19], position [20], vibration [21,22,23], velocity [24,25,26], liquid volume [27,28], and various chemicals in different phases [29,30,31,32]. Generally, the operation of triboelectric sensors is based on contact electrification and electrostatic induction [33,34,35]; specifically, the triboelectric behavior is based on surface phenomena. Therefore, triboelectric behavior and the corresponding signals are affected by an external environment. When the sensors are exposed to an environment, triboelectric sensors can be contaminated by organic/inorganic contaminants and H_2_O from air. Especially, the H_2_O in air can be a serious issue for the functionality of triboelectric mechanical sensors. H_2_O in air can form a conductive layer on tribo-materials, and the accumulated surface charges can dissipate through the H_2_O layer [36], which means that triboelectric sensors in a high relative humidity can provide incorrect data. Therefore, various researchers have focused on the development of humidity-resistant triboelectric devices based on physical sealing [37,38], material engineering [39,40,41], and surface engineering [42].

Atomic layer deposition (ALD) is a thin film deposition technique developed for the nanofabrication of integrated circuit systems, and it has been intensively studied and applied to various applications [43,44,45]. ALD enables the precise control of film thickness to the Angstrom scale with excellent conformality based on its unique self-saturation surface reactions. ALD is utilized for the design of electronic devices such as metal–insulator–metal (MIM) capacitors [46,47] and metal–oxide–semiconductor field effect transistors (MOSFETs) [48,49]. Additionally, ALD can be utilized to form an anti-corrosion layer for stable solar-based water splitting [50,51]. The functionality of photoanodes is maintained and the ALD-processed layer can act as a passivation layer because of the extremely low thickness and high uniformity.

In this work, we demonstrated a novel moisture passivation technique to maintain the functionality of triboelectric mechanical sensors. Especially, we aimed to propose the humidity passivation technique for triboelectric mechanical sensors that utilize the human skin as a counter tribo-material. Therefore, we selected a negative tribo-material, which is capable of the ALD process. ALD-TiO_x_ was deposited on a negative tribo-material, polytetrafluoroethylene (PTFE). To determine the effective process cycles for enhanced moisture passivation capability, triboelectric behaviors of TiO_x_-deposited PTFE films were evaluated and their physical characteristics were analyzed. The moisture passivation was related to the characteristics of the TiO_x_ layer on PTFE films. In addition, the moisture resistance could be improved by approximately three times compared with the pristine PTFE film. We expect that our technique can provide a novel method for developing stable and reliable triboelectric mechanical sensors in highly humid conditions.

## 2. Materials and Methods

### 2.1. Deposition of TiO_x_ on PTFE Film

TiO_x_ was deposited on a tribo-material using ALD. Before depositing TiO_x_, the PTFE film was attached to an Al sheet with an area of 1.5 cm × 1.5 cm. The ALD process was then conducted after loading the prepared PTFE/Al sheet. TiO_x_ was deposited in a traveling wave-type reactor at a temperature of 150 °C. Nitrogen (N_2_) was utilized as the carrier and purging gas. The base pressure was 1.12 × 10^−1^ Torr and the purge gas flow was 100 sccm. The titanium (IV) isopropoxide (TTIP) precursor was pulsed for 5 s and the water (H_2_O) reactant was pulsed for 1 s. An amount of 100, 300, 500, and 2000 deposition cycles were conducted to define the optimal ALD cycle to improve moisture resistance.

### 2.2. Assembly of the TENG Device and Triboelectric Performance Measurement

The prepared TiO_x_-deposited PTFE film was utilized as a negative tribo-material. An Al sheet was selected as a positive tribo-material. A pushing tester (JIOCS-120, Junil Tech Co., Deagu, Korea) was utilized to induce a compressive load on the TENG device. The measurement conditions for all samples were: a gap distance of 4 mm, a compressive load of 5 N, a contact frequency of 3 Hz, and a contact area of 1.5 cm × 1.5 cm. Electrical outputs were measured using an oscilloscope (MDO3052, Tektronix, Beaverton, OR, USA), a low-noise current preamplifier (SR570, Stanford Research Systems, Sunnyvale, CA, USA), and an electrometer (6514 system electrometer, Keithley, Solon, OH, USA). For evaluation of the humidity resistance, samples were stored in a humid chamber with a relative humidity (RH) of 99% for 24 h and the triboelectric performance was evaluated. To compare the signal loss rate, the initial triboelectric performance was measured at RH 10% and 25 °C.

### 2.3. Characterization

A field emission scanning electron microscope (FE-SEM, SU-70, Hitachi, Tokyo, Japan) was utilized to confirm the formation of TiO_x_ on PTFE with different process cycles. The water contact angle (WCA) of pristine PTFE and TiO_x_-deposited PTFE (TiO_x_/PTFE) films was measured using a droplet analyzer (SmartDrop, FemtoBioMed, Seongnam, Korea). Changes in the electronic structures were investigated using X-ray absorption spectroscopy (XAS). XAS experiments were performed at the 2 A beamline in the Pohang Accelerator Laboratory (PAL), Pohang, Korea. Surface chemical bonds were investigated, utilizing an X-ray photoelectron spectrometer (XPS, K-Alpha, Thermo Fisher Scientific Co., Waltham, MA, USA) with a pass energy of 20 eV and using a monochromatic Al Kα source. Survey scans were conducted to investigate the overall change of surface chemical bonds. After the survey scan, a narrow scan was performed. The C 1*s* and Ti 2*p* peaks were observed in PTFE and TiO_x_/PTFE samples. The narrow scan results were normalized and deconvoluted to compare the chemical bonds after depositing TiO_x_ and exposing it to humid air for 24 h. Finally, deconvolution of the narrow scan result was conducted to define the present chemical bonds and their portion.

## 3. Results and Discussion

### 3.1. Characteristics of ALD-TiO_x_ on PTFE Film

**Figure 1:**
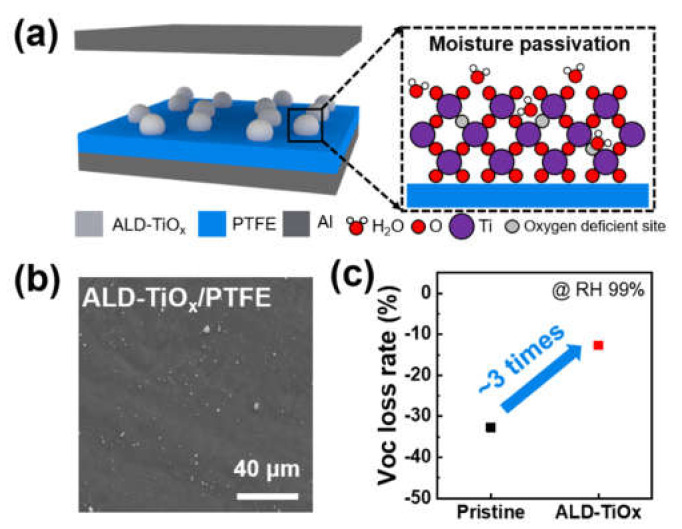
Concept and effectiveness of humidity passivation using ALD-TiO_x_. (**a**) Schematic illustration of humidity passivation using ALD-TiO_x_. (**b**) FE-SEM image of the ALD-processed TiO_x_/PTFE film. (**c**) Open circuit voltage (V_OC_) loss rate of pristine PTFE and ALD-TiO_x_-deposited PTFE film at relative humidity (RH) of 99%.

Figure 1 shows the concept of moisture passivation using TiO_x_ formed by ALD and the moisture passivation capability of the ALD TiO_x_/PTFE film. As shown in Figure 1a, the TiO_x_ layer was deposited on a PTFE surface. ALD-TiO_x_/PTFE film acts as a negative tribo-material and the aluminum (Al) sheet acts as both an electrode and positive tribo-material. The moisture passivation mechanism is based on the H_2_O absorption by oxygen-deficient sites. The FE-SEM images confirm that the TiO_x_ layer was formed on the PTFE surface in 300-ALD process cycles, as shown in Figure 1b. After depositing a TiO_x_ layer on PTFE, the moisture passivation capability with a pristine PTFE film was evaluated, as shown in Figure 1c. At the environmental humidity of RH 99%, pristine PTFE lost 32.7% of its triboelectric voltage signal. However, ALD-TiO_x_/PTFE film showed better moisture stability, which was approximately three times greater than pristine PTFE film. The corresponding voltage signal was reduced approximately 12.6% compared with the dry condition. To understand the better humidity stability of the ALD-TiO_x_/PTFE film, the formation and physical characteristics of the ALD-TiO_x_ layer were first analyzed.

**Figure 2:**
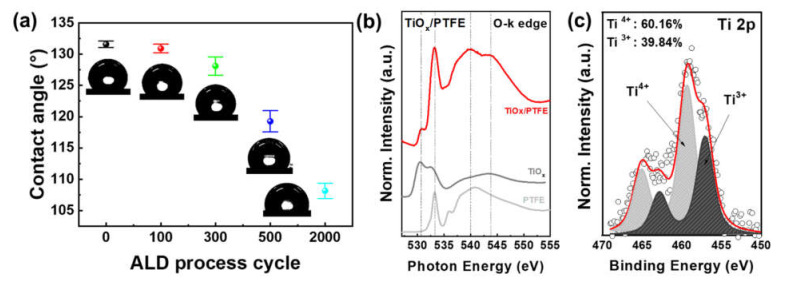
Effects of the ALD-TiO_x_ layer on the surface property. (**a**) Changes in the contact angle with an increase in number of ALD cycles. (**b**) Oxygen-κ edge XAS spectra of pristine PTFE (light gray), TiOx (gray), and ALD-TiO_x_/PTFE films (red). (**c**) Deconvoluted Ti 2*p* XPS spectrum of the ALD-TiO_x_ layer with 300 ALD cycles.

The surface of the ALD-TiO_x_/PTFE film was examined by the water contact angle (WCA) measurement. The average WCA value and standard deviation are presented in Figure 2a. The WCA of the pristine PTFE surface was ca. 131.6° and there was no significant change in WCA after depositing TiO_x_ with 100 ALD cycles (100-TiO_x_) (ca. 130.9°). As the number of ALD cycles increased to 300 cycles (300-TiO_x_), the contact angle decreased to 128.1° because of the formation of the TiO_x_ layer. The WCA rapidly decreased to 119.3° and 108.2° after 500 ALD cycles (500-TiO_x_) and 2000 ALD cycles (2000-TiO_x_), respectively. The change in WCA is related to the distribution of the TiO_x_ layer. Figure S1 indicates the top-view FE-SEM images with EDS mapping of the Ti distribution. As shown in Figure S1a, there were no Ti atoms on the pristine PTFE film. It was difficult to identify Ti atoms from the 100-TiO_x_/PTFE film, similar to the pristine PTFE film (Figure S1b). However, from 300- to 500-TiO_x_/PTFE films, irregular island-shaped TiO_x_ particles were observed (Figure S1c,d). This island-growth is related to the TiO_x_ nucleation on the PTFE surface. When the ALD process is conducted on polymeric materials, the C=O bonds act as nucleation sites for ALD growth [52]. However, an ideal PTFE film has only C_2_F_4_ bonds, resulting in difficult TiO_x_ nucleation on a PTFE surface without surface treatments such as plasma etching. In this work, we utilized an industrial PTFE film with a purity of 99.9%. Therefore, the TiO_x_ nucleation might progress at impurities in the PTFE film. In a 2000-TiO_x_/PTFE film, a TiO_x_ film was formed on the PTFE (Figure S1e). As a result, the TiO_x_ layer was grown from island to film as the ALD cycle increased. Thus, we assumed that the triboelectric performance would proportionally decrease with an increase in ALD cycles because of the transition of tribo-materials from PTFE to TiO_x_. Figure 2b indicates the normalized oxygen k-edge XAS spectra of pristine PTFE (light gray line), TiO_x_ (gray line), and 300-TiO_x_/PTFE composite surface (red line). TiO_x_ has a peak at a photon energy of 531 eV and PTFE has a peak at 533 eV. In the hybridized spectrum, noticeable peaks from TiO_x_ and PTFE were observed, which means that the TiO_x_ and PTFE co-exist at the surface when the ALD process is conducted with a relatively low ALD cycle. In addition, the XAS spectra show that the dominant surface material of the 300-TiO_x_/PTFE film is PTFE. Therefore, we expected that the triboelectric performance of hybridized surface might be similar to that of pristine PTFE. The chemical bonding state of the TiO_x_ layer with Ti 2*p* spectrum was evaluated, as shown in Figure 2c. The Ti 2*p* spectrum of 300-TiO_x_ was deconvoluted, and different Ti states of Ti^4+^ (60.16%) and Ti^3+^ (39.84%) existed in the TiO_x_ layer. The O 1*s* spectrum of 300-TiO_x_ was deconvoluted as well, as indicated in Figure S2a. The deconvoluted O 1*s* spectrum also designates the presence of Ti^3+^. In the 500-TiO_x_ film, there were two different states, as shown in Figure S3(ai), 56.7% Ti^4+^ and 46.3% Ti^3+^. Therefore, the humidity passivation might be related to the oxygen-deficient sites in the ALD-TiO_x_ layer.

### 3.2. Triboelectric Behavior of the ALD-TiO_x_/PTFE Film

**Figure 3:**
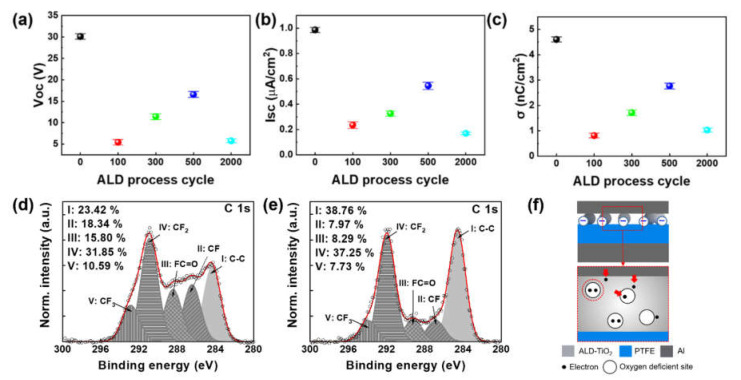
Effects of the ALD-TiO_x_ layer on triboelectric outputs. Changes in (**a**) the open circuit voltage (V_OC_), (**b**) short circuit current (I_SC_), and (**c**) charge density (σ) as a function of ALD cycles. Deconvoluted C 1*s* XPS spectra of (**d**) pristine PTFE film and (**e**) 300-TiO_x_/PTFE film. (**f**) Schematic illustration of the electron trap mechanism according to the oxygen deficient sites.

After confirming the existence and formation of TiO_x_/PTFE composite surfaces, their triboelectric behaviors were evaluated, as shown in Figure 3. Figure 3a–c show the measured open circuit voltage (V_OC_), short circuit current (I_SC_), and charge density (σ) at RH 10% and 25 °C. The overall triboelectric performances decreased with the deposition of TiO_x_ layers. Pristine PTFE produced a V_OC_ of 30.1 V and an I_SC_ of 0.98 μA, respectively, and its surface charge density was 4.61 nC/cm^2^. However, the triboelectric performances significantly decreased with the 100-TiO_x_/PTFE film. The measured V_OC_, I_SC_, and σ values were 5.4 V, 0.24 μA, and 0.81 nC/cm^2^, respectively. The the 300-TiO_x_/PTFE film produced larger triboelectric signals than the 100-TiO_x_/PTFE film. The generated V_OC_, I_SC_, and σ were 11.4 V, 0.33 μA, and 1.7 nC/cm^2^, respectively. The 500-TiO_x_/PTFE film showed the highest performance among the ALD-TiO_x_-deposited PTFE films in this work. The detected average triboelectric signals were 16.6 V, 0.55 μA, and 2.8 nC/cm^2^, respectively. Finally, the triboelectric performance of a 2000-TiO_x_/PTFE film was evaluated. The generated V_OC_ and I_SC_ were 5.8 V and 0.17 μA, respectively, and the accumulated charge was 1.0 nC/cm^2^, which is slightly greater than that of the 100-TiO_x_/PTFE sample. Furthermore, the mechanical durability of ALD-TiO_x_/PTFE film (300-TiO_x_/PTFE) was assessed to ensure the stability of the ALD-TiO_x_ layer, as shown in Figure S4. The ALD-TiO_x_/PTFE film showed a constant output signal after 60,000 contact/separation trials over 5 h. This result shows that the ALD-TiO_x_ layer will be maintained under the mechanical stimulation.

After confirming the triboelectric signals of ALD-TiO_x_/PTFE films, we tried to understand the cause of electrical output attenuation. The output reduction in the 2000-TiO_x_/PTFE film can be explained based on the FE-SEM and EDS results in Figure S1e. After conducting the ALD process with 2000 cycles, the PTFE surface was completely covered by TiO_x_ thin film, which means that the triboelectric material changed to a TiO_x_ thin film. TiO_x_ is a relatively positive tribo-material compared with PTFE. Therefore, the reduction in the triboelectric performance is inevitable when the TiO_x_ film is formed on the PTFE surface. Figure 3d,e show the deconvoluted C 1*s* spectra of a pristine PTFE and 300-TiO_x_/PTFE film. As shown in Figure 3d, pristine PTFE film has various chemical bonds, such as C–C, CF, CF_2_, CF_3_, and FC=O. As expected, there was a FC=O bond, which can act as a nucleation site during the ALD process. After 300 ALD cycles, there were changes in the chemical bonding states, as indicated in Figure 3e. Originally, the chemical bonding states, which were bonded with fluorine atoms, were 76.58% in the pristine PTFE. However, after forming the TiO_x_ layer, the portion of detected fluorine decreased to 61.24%, which is an approximately 15% decrease. In addition, the portion of FC=O bonds decreased from 15.8% to 8.29%, which indirectly illustrates that the TiO_x_ nucleation progressed in this chemical bond. The reduction in chemical bonding states containing fluorine also indicates the transition of the triboelectric surface from PTFE to TiO_x_. Therefore, the reduction in the triboelectric performance is related to changes in the triboelectric property of the PTFE surface. However, when the coverage of the TiO_x_ layer at each ALD process is considered, the triboelectric performance of the 100-TiO_x_ sample should be greater than the other ALD processed samples. The largest signal loss in the 100-TiO_x_ sample is based on the possible physical phenomenon during the ALD process. During the ALD process, the PTFE surface is exposed to two different atmospheres consisting of two different precursors, TTIP and H_2_O, at a relatively high temperature (150 °C). When handling the polymers at a certain temperature, the glass transition temperature should be considered. At the glass transition temperature, the rigidity and viscosity of the polymer decrease, the fluidity of the solid increases and the molecular motion intensifies [53]. The glass transition temperature of PTFE is approximately 120 °C [54], which means that during the ALD process, the PTFE polymer chain is relatively released. A relaxed PTFE chain makes more space for atoms or molecules, and it enables the precursor molecules to infiltrate the PTFE film. Therefore, while the PTFE layer thermally deforms, the precursor molecules can infuse into the PTFE layer [55]. Diffused precursor molecules can deflect the electrostatic induction caused by surface charges on the composite surface and can reduce the corresponding triboelectric performance. As the ALD cycle is increased, the amount of diffused precursors may increase. However, TiO_x_ nanoparticles are able to capture electrons in oxygen-deficient sites [56]. The oxygen-deficient sites are electrically positive; therefore, they can act as electron trapping sites. Figure 3f shows the proposed mechanism for enhancing the triboelectric performance of ALD-TiO_x_/PTFE TENGs. As both PTFE and TiO_x_ particles co-exist with relatively low ALD cycles (from 100 to 500 cycles), contact electrification with the Al sheet occurs in both PTFE and ALD-TiO_x_ particles. When the Al sheet is in contact with the TiO_x_ particles, surface charges are formed on TiO_x_ particles. Additionally, the free electrons in the Al sheet can be trapped in oxygen-deficient sites in TiO_x_. The trapped electrons can compensate for the initial surface charge loss induced by changes in the contact material from PTFE to TiO_x_. By increasing the subsequent ALD process cycles, which lead to the uniform distribution of TiO_x_ particles, the possibility of electron trapping could be improved. Therefore, the signal loss from changes in tribo-material and electrostatic induction could be compensated.

### 3.3. Moisture Passivation of TiO_x_ and Triboelectric Behaviors

**Figure 4:**
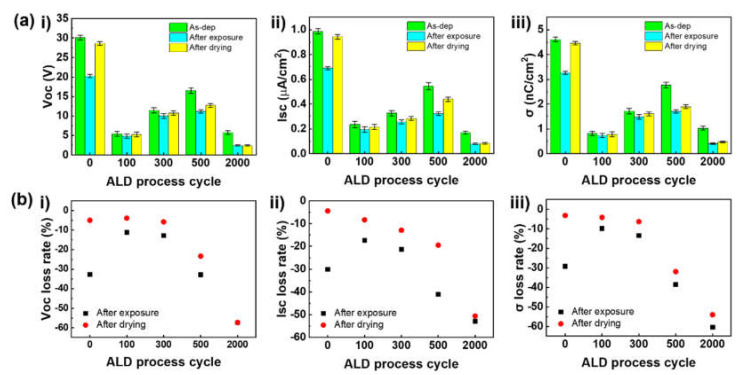
Effect of moisture on the triboelectric outputs of ALD-TiO_x_/PTFE films. (**a**) Comparison of triboelectric signals after moisture exposure and natural drying process. (**b**) Triboelectric signal loss rate of ALD-TiO_x_/PTFE films after moisture exposure and drying process as a function of ALD cycles; (i) open circuit voltage (V_OC_), (ii) short circuit current (I_SC_), and (iii) charge density (σ).

**Figure 5:**
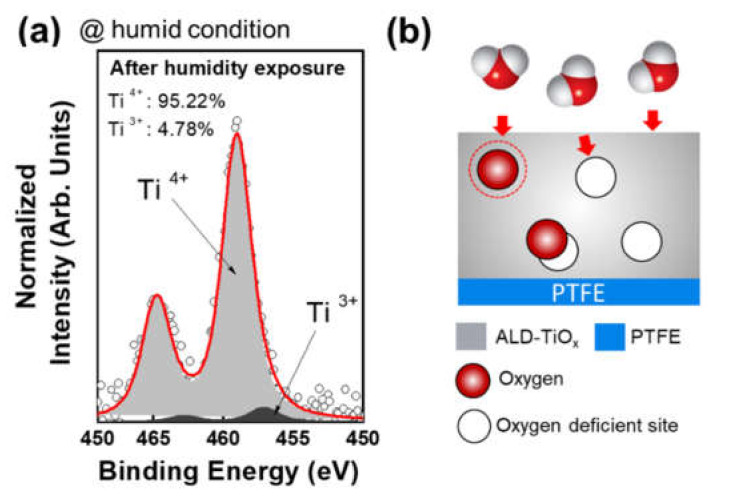
Change in the chemical bonding state after humidity exposure and relevant mechanism. (**a**) Deconvoluted Ti 2*p* spectrum after moisture exposure with RH 99% at room temperature. (**b**) Schematic illustration of humidity passivation mechanism according to the oxygen deficient sites.

After confirming the base triboelectric behaviors of the ALD-TiO_x_/PTFE films, the humidity stabilities were evaluated. For this evaluation, all samples were contained in an environment-controlled chamber (RH 99% and room temperature) for 24 h. The triboelectric performances were measured immediately upon removal from the chamber. Furthermore, the triboelectric performances of the ALD-TiO_x_/PTFE films were evaluated to confirm their reusability after the natural drying process at room temperature, as shown in Figure 4 and Figure S5. Figure 4(ai–aiii) show the measured open circuit voltage (V_OC_), short circuit current (I_SC_), and charge density (σ), respectively. The light green bars indicate the triboelectric behaviors of the as-deposited ALD-TiO_x_/PTFE films. The light blue bars represent the average triboelectric performances after the humidity exposure. Yellow bars show the average triboelectric performance after the natural drying process. After humidity exposure, the pristine PTFE film produced a voltage of 20.3 V and a current of 0.69 μA, and the accumulated charges were 3.26 nC/cm^2^. The output loss rate was calculated and is displayed in Figure 4(bi–biii). The black dots indicate the output loss rate after humidity exposure and red dots show the signal loss rate after the natural drying process. Compared with the triboelectric performances at the dry condition (As-dep), the overall output decreased approximately 30%. Interestingly, the 100-TiO_x_/PTFE film showed better passivation capability than the pristine PTFE film. The 100-TiO_x_/PTFE film generated a voltage of 5.4 V and current of 0.19 μA, with accumulated charges of 0.73 nC/cm^2^. Reduction rates of the voltage, current, and charge density were 11%, 17.4%, and 9.7%, respectively. The reduction rates were lower than in the pristine PTFE film. The 300-TiO_x_/PTFE film produced a voltage of 10 V and a current of 0.26 μA, respectively. The accumulated charges were 1.5 nC/cm^2^. The voltage was reduced approximately 12.7%, the current was reduced approximately 21%, and the charge density decreased approximately 13% compared with the triboelectric outputs of 300-TiO_x_/PTFE film in the dry condition. However, the 500-TiO_x_/PTFE film shows a relatively poor moisture passivation ability compared with the 100 and 300-TiO_x_/PTFE films. A σ of 1.7 nC/cm^2^ was formed on the 500-TiO_x_/PTFE film, and the corresponding V_OC_ and I_SC_ were 11.1 V and 0.32 μA, respectively. The signal loss rates of V_OC_, I_SC_, and σ were 32.9%, 41%, and 38%, respectively. The signal loss rates of the 500-TiO_x_/PTFE films were less than the pristine PTFE film, indicating that the ALD process longer than 500 cycles did not improve the moisture passivation. The 2000-TiO_x_/PTFE film shows the lowest moisture passivation capability among the ALD-TiO_x_/PTFE samples. After the humidity exposure, the 2000-TiO_x_/PTFE film produced a V_OC_ of 2.5 V, an I_SC_ of 0.08 μA, and measured σ of 0.4 nC/cm^2^. The V_OC_ signal dropped by approximately 57%, the I_SC_ signal was reduced approximately 53%, and σ decreased by approximately 60%. The moisture passivation mechanism can be explained based on the role of the oxygen-deficient site and hydrophobicity of ALD-TiO_x_/PTFE films. The oxygen-deficient site can absorb oxygen ions [56]. Figure 5a shows the Ti 2*p* spectrum of the 300-TiO_x_/PTFE film after humidity exposure. Compared with the Ti 2*p* spectrum in Figure 2c, the portion of oxygen-deficient sites (Ti^3+^) decreased from 39.14% to 4.78%. As shown in Figure S2b, the O 1*s* spectrum of the 300-TiO_x_/PTFE film also designates the decrease in oxygen-deficient sites. The TiO_x_ layer can absorb the H_2_O from air and can protect the PTFE surface from moisture in the environment, as shown in Figure 5b. As shown in Figure S3a, in the 500-TiO_x_/PTFE film, there was 43.3% Ti^3+^ and a decrease to 11% after humidity exposure. Therefore, this passivation mechanism is also valid for explaining the triboelectric behavior of 500-TiO_x_/PTFE film. In addition to the absorption of H_2_O, the oxygen-deficient site can trap the electrons. However, after the oxygen-deficient site absorbs H_2_O from the air, the possibility of electron trapping can decrease. Therefore, it is difficult to obtain charge compensation by electron trapping. Therefore, 500-TiO_x_/PTFE, which has a large amount of Ti^3+^, can have a relatively larger signal loss than the 100 and 300-TiO_x_/PTFE films. The TiO_2_ film is grown after 2000 ALD cycles, as shown in Figures S1e and S3b. Therefore, H_2_O trapping-based passivation is more difficult than particle TiO_x_/PTFE films. Furthermore, TiO_2_ is a common hydrophilic material. Thus, H_2_O molecules can easily bind the TiO_x_ layer and form a thin conductive layer, which can dissipate the surface charges and reduce the triboelectric performance. 

After confirming the moisture passivation mechanism, we measured triboelectric performances after a natural drying process at room temperature for 24 h. The triboelectric performance of PTFE was noticeably recovered after natural drying. The measured V_OC_, I_SC_, and σ were 28.6 V, 0.94 μA, and 4.46 nC/cm^2^, respectively. The recovered triboelectric signals and charge density were the same as approximately 96% of outputs at the dry condition. However, the ALD-TiO_x_/PTFE films show a relatively low recovery rate compared with the pristine PTFE film. Triboelectric performances of 100-TiO_x_/PTFE recovered by approximately 5 to 8% (91–96% of outputs at the dry condition) and the corresponding V_OC_ and I_SC_ were 5.2 V and 0.22 μA. The surface charge density was 0.78 nC/cm^2^. The 300-TiO_x_/PTFE showed a similar recovery rate, 6 to 9% (87–94% of performance at the dry condition), as much as that of the 100-TiO_x_/PTFE. The accumulated charge was 1.6 nC/cm^2^ and the detected open circuit voltage and short circuit current were 10.2 V and 0.28 μA, respectively. The 500-TiO_x_/PTFE film produced a V_OC_ of 12.7 V and I_SC_ of 0.44 μA, which were 9 and 22% recovered signals (68–80% of the performance in the dry condition), respectively. The charge density was 1.9 nC/cm^2^, which was recovered to approximately 7%. The output signals of the 2000-TiO_x_/PTFE film was barely recovered to approximately 0.1 to 6% (42–49% of the performance at the dry condition). The detected surface charge was 0.47 nC/cm^2^ and the corresponding V_OC_ and I_SC_ were 2.46 V and 0.08 μA, respectively. The output recovery rates indicate the hysteresis of the triboelectric performance. The hysteresis of triboelectric outputs is related to the TiO_x_ transformation to TiO_2_ as well as the presence of a thin H_2_O layer. To completely recover the triboelectric performance after the natural drying process, the absorbed oxygen has to break and an ALD-deposited layer needs to transform to the TiO_x_ layer. However, TiO_2_ is a stable chemical state, and it is difficult to break with energy at room temperature. Thus, original oxygen-deficient sites can still be occupied with H_2_O absorbed during humidity exposure process. A decrease in oxygen-deficient sites leads to the low recovery rate of ALD-TiO_x_/PTFE films. In addition, because of the increased hydrophilicity of the TiO_x_ layer, it is possible that the H_2_O layer could remain on the TiO_x_ layer. Thus, the remaining H_2_O layer can dissipate the surface charges. Therefore, the corresponding triboelectric outputs of the 2000-TiO_x_/PTFE film could be limited after the natural drying process. Our moisture passivation technique can decrease the peak triboelectric signal because of the transition of the tribo-material and diffusion of precursors during the ALD process. However, the technique can improve the moisture stability of PTFE by approximately three times. Thus, we expect that our moisture passivation technique can be utilized to preserve the accuracy of triboelectric sensors in a highly humid environment.

## 4. Conclusions

In this work, we proposed a novel moisture passivation technique using a TiO_x_ layer deposited by the ALD for triboelectric mechanical sensors. The TiO_x_ layer on a PTFE film was investigated by 100, 300, 500, and 2000 ALD process cycles. At a few ALD process cycles from 100 to 500, TiO_x_ layers were grown as islands because of the ALD nucleation mechanism on the polymer surfaces. At the most ALD cycles (2000 ALD process cycles), the ALD-TiO_x_ layer was grown as a uniform film on a PTFE film. Due to the transformation of tribo-materials from PTFE to TiO_x_/PTFE, the base triboelectric signals were reduced over 50%. However, oxygen-deficient sites in the TiO_x_ layer compensate the tribo-material transition caused surface charge loss by trapping electrons in the Al electrode. ALD-TiO_x_ grown with few ALD cycles shows better moisture stability and triboelectric signal at humid conditions, which are the same as 90% of outputs at the dry condition, while pristine PTFE generated 70% of outputs at the dry condition. The improved moisture stability is based on the H_2_O absorption by an oxygen-deficient site in the TiO_x_ layer. Due to the improved moisture stability, the triboelectric mechanical sensors can preserve the fine sensing resolution at the highly humid environment. We expected that our approach can be used to maintain the triboelectric signal of triboelectric mechanical sensors in harsh environments.

## Supplementary Materials

The following are available online at https://www.mdpi.com/article/10.3390/nano11071795/s1, Figure S1: FE-SEM and EDS elemental mapping of Ti on ALD-TiOx/PTFE films, Figure S2: deconvoluted XPS O 1s spectra of 300 ALD-TiOx/PTFE film, Figure S3: deconvoluted XPS Ti 2p spectra of 500 and 2000 ALD-TiOx/PTFE films, Figure S4: mechanical durability test, Figure S5: measured triboelectric signals at the dry condition, at highly humid condition, and after the natural drying process.
