# Supplementary material for: Toward Enhanced Humidity Stability of Triboelectric Mechanical Sensors via Atomic Layer Deposition
Source: Nanomaterials (Basel). 2021 Jul 9;11(7):1795. doi: 10.3390/nano11071795 (PMC8308376; doi:10.3390/nano11071795)
Supplement: Supplementary file 1 [file nanomaterials-11-01795-s001.zip › nanomaterials-1261208-supplementary.pdf]

Supplementary Materials

Toward Enhanced Humidity Stability of Triboelectric Mechanical Sensors via Atomic Layer Deposition

Wook Kim [1,†], Sumaira Yasmeen [2,†], Chi Thang Nguyen [2], Han-Bo-Ram Lee [2,*] and Dukhyun Choi [1,*]

[1] Department of Mechanical Engineering (Integrated Engineering Program), Kyung Hee University, Yongin 17104, Korea; choice124@khu.ac.kr
[2] Department of Materials Science and Engineering, Incheon National University, Incheon 22012, Korea; sumairayasmeen51@gmail.com (S.Y.); victornguyen@inu.ac.kr (C.T.N.)
* Correspondence: hbrlee@inu.ac.kr (H.-B.-R.L.); dchoi@khu.ac.kr (D.C.)
† These authors equally contributed to this work.

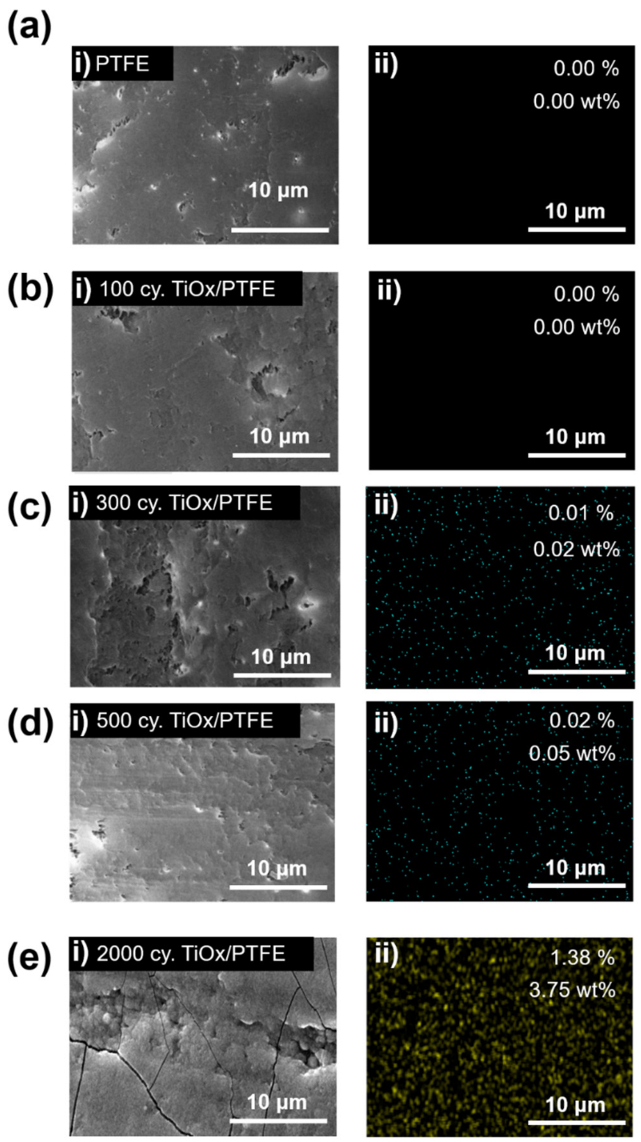

Figure S1. FE-SEM and EDS elemental mapping of Ti on (**a**) pristine PTFE film and ALD-TiO_x/PTFE films grown with (**b**) 100, (**c**) 300, (**d**) 500, and (**e**) 2000 ALD cycles. i) FE-SEM image in top view and ii) EDS elemental mapping of Ti on ALD-TiO_x/PTFE films.

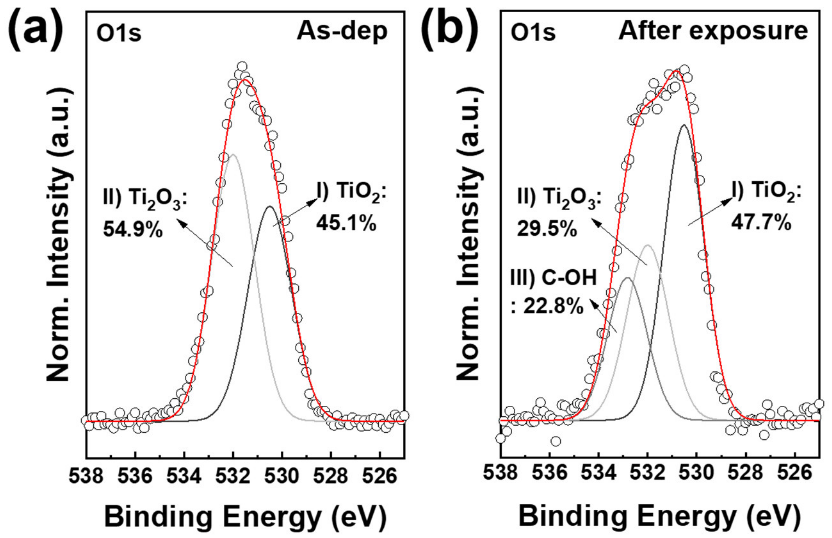

Figure S2. Deconvoluted XPS O 1*s* spectra of 300-TiO_x/PTFE at (**a**) dry (RH 10%) and (**b**) humid conditions (RH 99%).

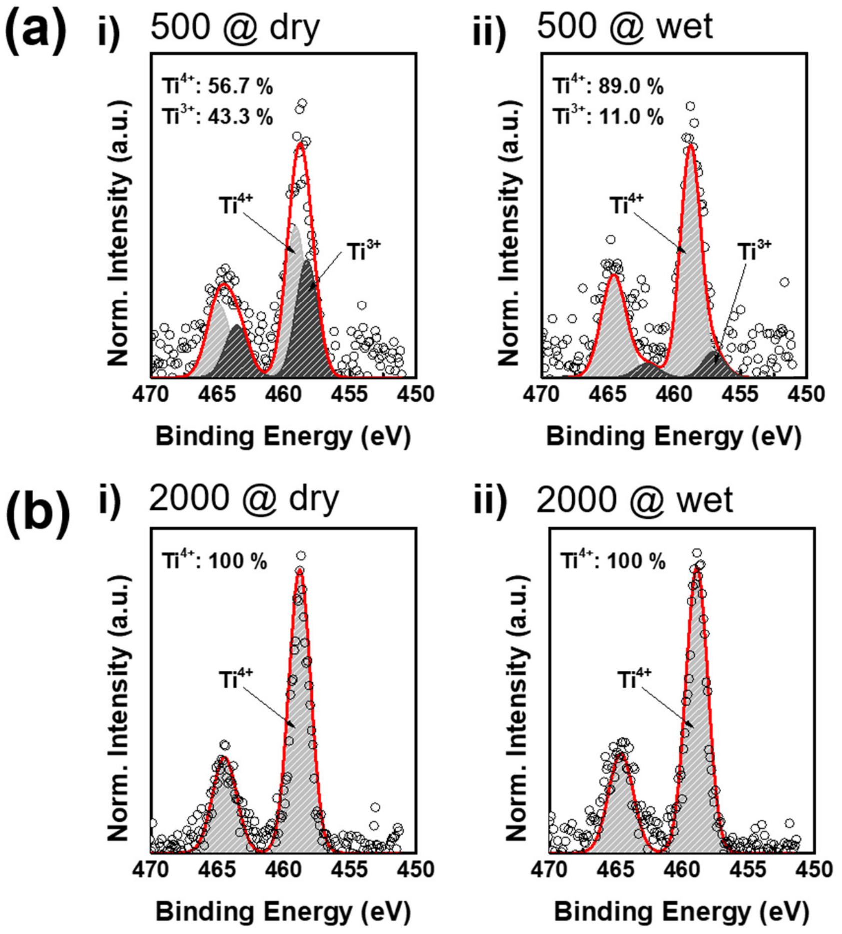

Figure S3. Deconvoluted XPS Ti 2*p* spectra of (**a**) 500-TiO_x/PTFE and (**b**) 2000-TiO_x/PTFE films. Ti 2*p* spectrum of the i) as-deposited specimen and ii) after moisture exposure.

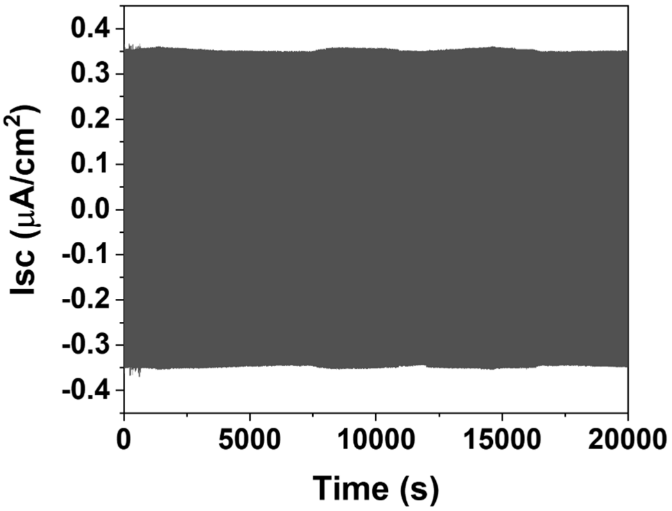

Figure S4. Mechanical durability test of 300-TiO_x/PTFE film under the cyclic mechanical stimulation for 20,000 s. To evaluate the mechanical stability, the contact load of 5 N and contact frequency of 3 Hz were applied.

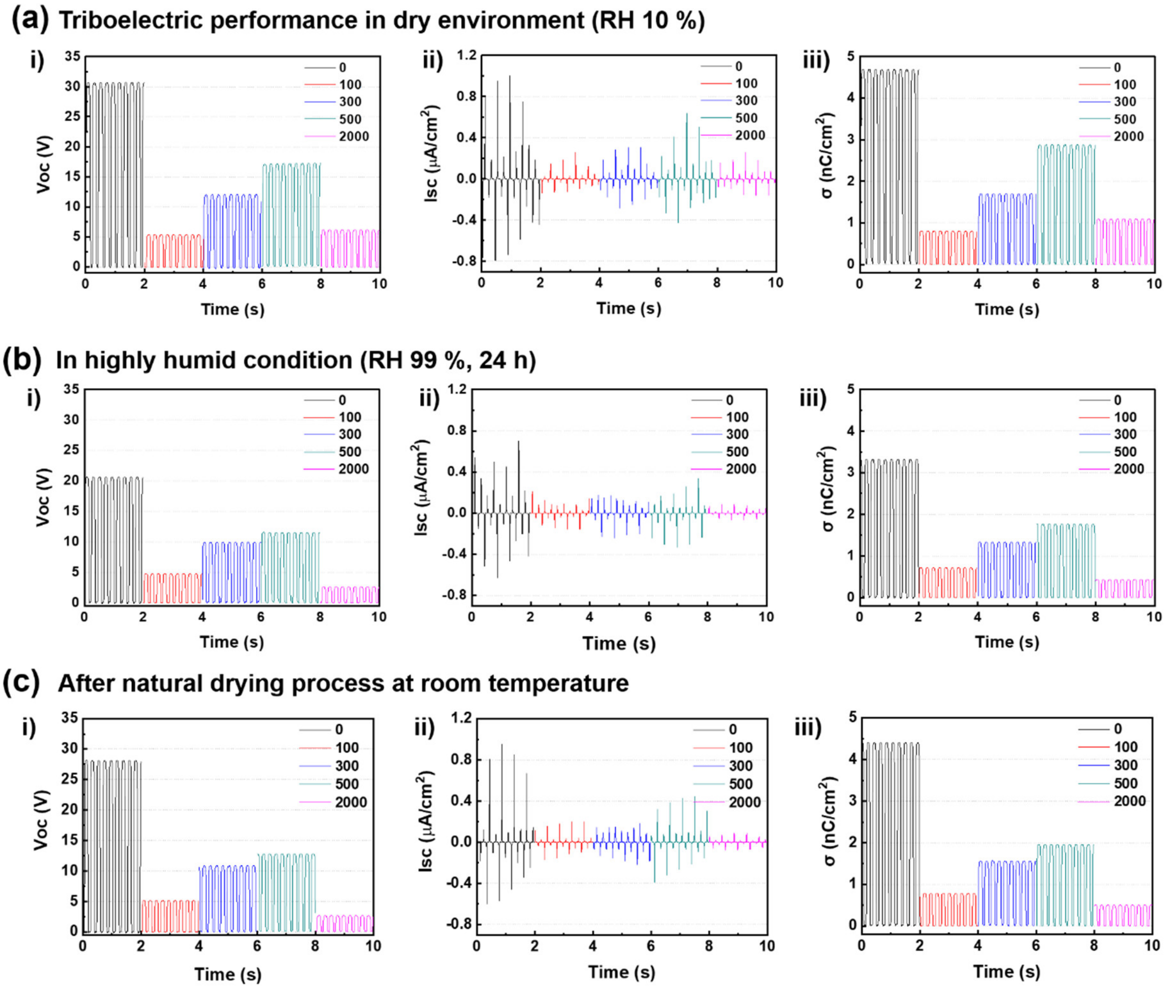

Figure S5. Measured triboelectric outputs of ALD-TiO_x/PTFE films in (**a**) dry (RH 10%) and (**b**) humid environments (RH 99%). (**c**) Triboelectric signals after a natural drying process: i) open circuit voltage, ii) short circuit current, and iii) charge density.
